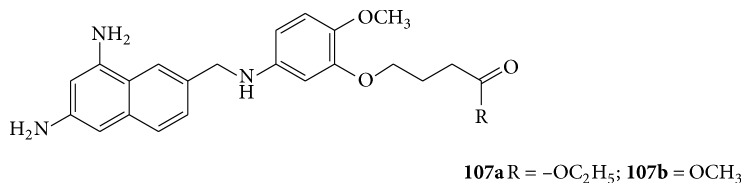# Chemical Characteristics, Synthetic Methods, and Biological Potential of Quinazoline and Quinazolinone Derivatives

**DOI:** 10.1155/2014/395637

**Published:** 2014-11-12

**Authors:** Mohammad Asif

**Affiliations:** Department of Pharmacy, GRD (PG) Institute of Management and Technology, Dehradun, Uttarakhand 248009, India

## Abstract

The heterocyclic fused rings quinazoline and quinazolinone have drawn a huge consideration owing to their expanded applications in the field of pharmaceutical chemistry. Quinazoline and quinazolinone are reported for their diversified biological activities and compounds with different substitutions bring together to knowledge of a target with understanding of the molecule types that might interact with the target receptors. Quinazolines and quinazolinones are considered as an important chemical for the synthesis of various physiological significance and pharmacological utilized molecules. Quinazolines and quinazolinone are a large class of biologically active compounds that exhibited broad spectrum of biological activities such as anti-HIV, anticancer, antifungal, antibacterial, antimutagenic, anticoccidial, anticonvulsant, anti-inflammatory, antidepressant, antimalarial, antioxidant, antileukemic, and antileishmanial activities and other activities. Being considered as advantaged scaffold, the alteration is made with different substituent.

## 1. Introduction

**Figure 1:**
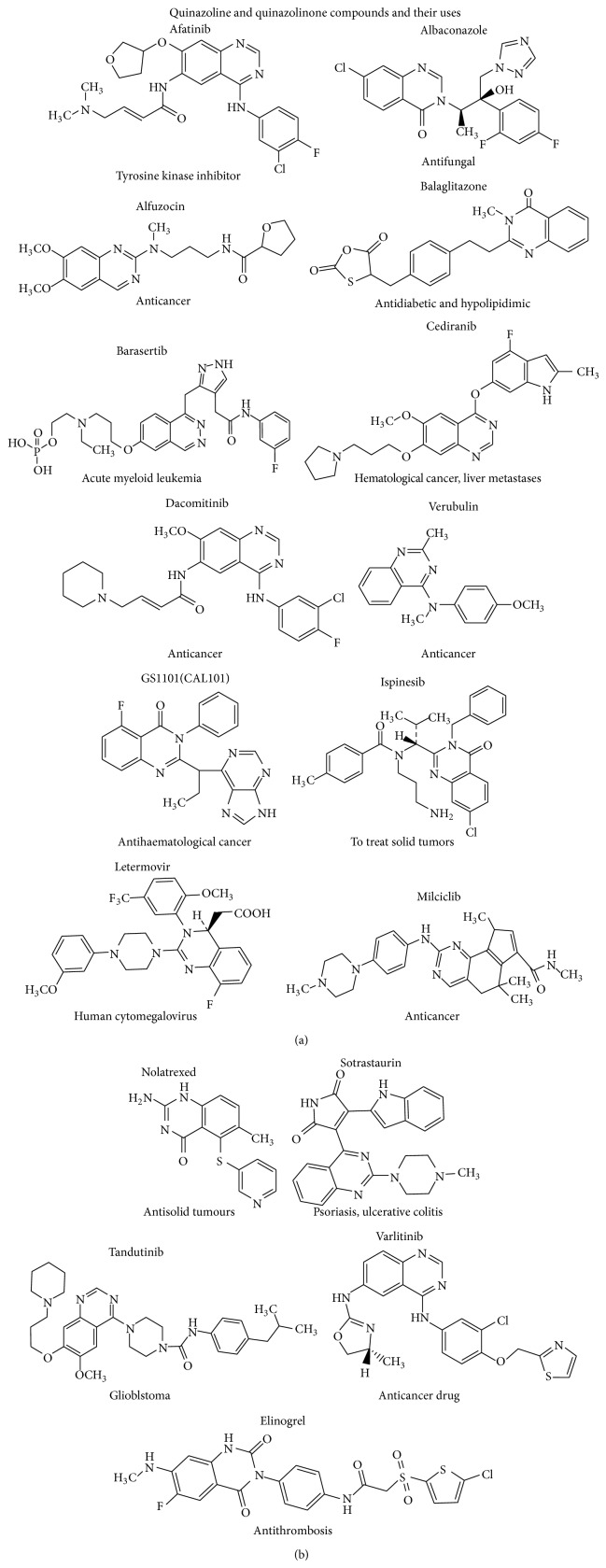
Some marketed available drugs contain quinazoline and quinazolinone moiety [2].

Quinazolines and quinazolinones are classes of fused heterocycles that are of considerable interest because of the diverse range of their biological properties [1]. Many substituted quinazoline and quinazolinone derivatives possess a wide range of bioactivities such as antimalarial, anticancer, antimicrobial, antifungal, antiviral, antiprotozoan, anti-inflammatory, diuretic, muscle relaxant, antitubercular, antidepressant, anticonvulsant, acaricidal, weedicide, and many other biological activities. Quinazoline and quinazolinone compounds are also used in preparation of various functional materials for synthetic chemistry and also present in various drugs molecules (Figure 1). This review is an attempt to expand the huge potentiality and focused on the various biological activities of quinazolines and quinazolinones [2].

**Scheme 1:**
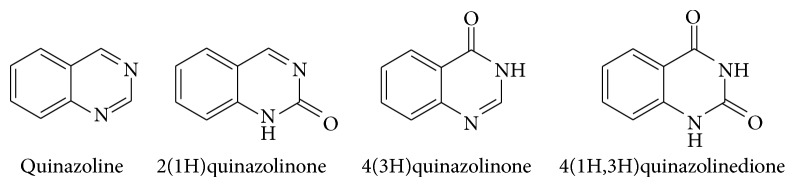


Quinazolinones will be classified into the following five categories, based on the substitution patterns of the ring system [3]. These are 2-substituted-4(3H)-quinazolinones, 3-substituted-4(3H)-quinazolinones, 4-substituted-quinazolines, 2,3-disubstituted-4(3H)-quinazolinones, and 2,4-disubstituted-4(3H)-quinazolinones. Depending upon the position of the keto or oxo group, these compounds may be classified into three types [4]. Out of the three (2(1H)quinazolinones, 4(3H)quinazolinones and 2,4(1H,3H)quinazolinedione) quinazolinone structures, 4(3H)-quinazolinones are most prevalent, either as intermediates or as natural products in many proposed biosynthetic pathways (see Scheme 1).

This is partly due to the structure being derived from the anthranilates (anthranilic acid or various esters, isatoic anhydride, anthranilamide, and anthranilonitrile) while the 2(1H)-quinazolinone is predominantly a product of anthranilonitrile or benzamides with nitriles [4].

## 2. History

**Scheme 2:**
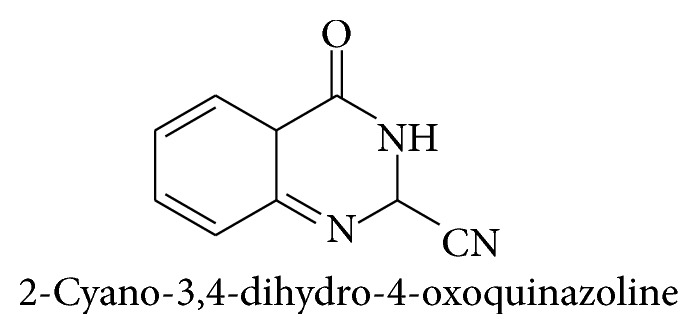


In 1869 Griess prepared the first quinazoline derivative, 2-cyano-3,4-dihydro-4-oxoquinazoline, by the reaction of cyanogens with anthranilic acid. The bicyclic product was called bicyanoamido benzoyl and used this name until 1885 [5]. The preparation of the quinazoline came many years later when Bischler and Lang obtained it by decarboxylation of the 2-carboxy derivative. A more satisfactory synthesis of quinazoline was subsequently devised by Gabriel in 1903. The name was proposed by Widdege. Other names such as phenmiazine, benzyleneamidine, benzo-1,3-diazine, 5,6-benzopyrimidine, and 1,3-diazanapthaline have occasionally been used. The presence of a fused benzene ring alters the properties of the pyrimidine ring considerably. The two nitrogen atoms are not equivalent, and the marked polarization of the 3,4-double bond is reflected in the reactions of quinazoline. The properties of substitute's quinazolines depend largely on (a) the nature of the substituents, (b) whether they are in the pyrimidine ring or in the benzene ring, and (c) whether or not complete conjugation is present in the pyrimidine ring [6–8] (see Scheme 2).

## 3. Chemical Properties of Quinazolines

The chemistry of quinazoline was reviewed by Williamson in 1957 and then by Lindquist in 1959 and brought up to date by Armarego in 1963.

Quinazolines is stable in cold dilute acid and alkaline solutions, but it is destroyed when these solutions are boiled. O-Aminobenzaldehyde, ammonia, and formic acid are formed when quinazoline is boiled with hydrochloric acid.

### 3.1. Hydrolysis, Oxidation, and Reduction

Oxidation of quinazoline in dilute aqueous acid with two equivalents of hydrogen peroxide at room temperature gave 3,4-dihydro-4-oxo quinazoline. In alkaline medium, the anhydrous neutral species of quinazoline were predominantly undergo oxidation with KMnO_4_ and yielded 3,4-dihydro-6 4-oxo quinazoline.

#### 3.1.1. Oxidation

**Scheme 3:**
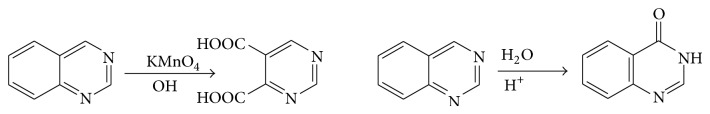


Catalytic hydrogenation of quinazoline stopped after the absorption of one molecule of hydrogen and gave 3,4-dihydro quinazoline (see Scheme 3).

#### 3.1.2. Reduction

**Scheme 4:**
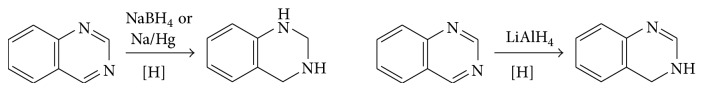


Reduction with sodium amalgam gave 1,2,3,4-tetrahydroquinazoline. Lithium aluminum hydride and sodium borohydride gave 3,4-dihydro and 1,2,3,4-tetrahydroquinazoline (see Scheme 4).

### 3.2. Nucleophilic and Electrophilic Substitution Reactions

**Scheme 5:**
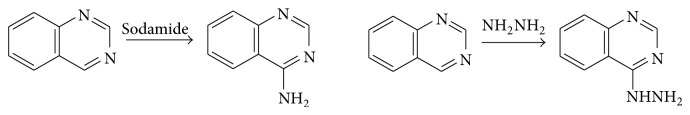


The two known nucleophilic substitution reactions of quinazoline are sodamide and hydrazine most probably proceed via the intermediate addition products, and gave 4-amino and 4-hydrazine quinazoline (see Scheme 5).

#### 3.2.1. Electrophilic Substitution Reaction of Quinazoline

**Scheme 6:**
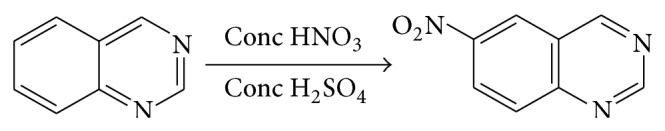


Nitration is the only known electrophilic substitution reaction of quinazoline. The expected order of reactivity is at positions 8 > 6 > 5 > 7 > 4 > 2. Quinazoline gives 6-nitroquinazoline with fuming nitric acid in concentrated H_2_SO_4_. No oxidation of the heterocyclic ring can occur under these conditions because the hydrated cation is not present (see Scheme 6).

### 3.3. Alkylation Reactions

**Scheme 7:**
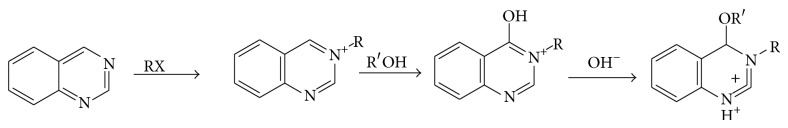


Alkylation of quinazoline takes place on N atom, 3-methyl, 3-ethyl-3-alkyl, and 3-benzylquinazolinium salts that readily take up a molecule of alcohol to form the corresponding 4-alkoxy-3-alkyl-3,4-dihydro quinazolinium salts. These salts gave the pseudo bases, 3-alkyl-3,4-dihydro-4-hydroxy quinazolines on treatment with strong alkali (see Scheme 7).

### 3.4. Addition Reactions

Quinazoline is highly reactive towards anionic reagents which attack on position 4. Sodium bisulphate, hydrogen cyanide, acetone, 2-butanone, acetophenone, and cyclohexanone add across the 3,4-double bond of quinazoline. Methyl, ethyl, isopropyl, benzyl, t-butyl, and phenyl magnesium halides and phenyl lithium also add across the 3,4-double bond to give the corresponding 4-substituted 3,4-dihydroquinazolines.

## 4. Synthesis of Quinazoline Compounds

Various methods were reported for the synthesis of oxoquinazolines.

### 4.1. Niementowski's Synthesis

**Scheme 8:**
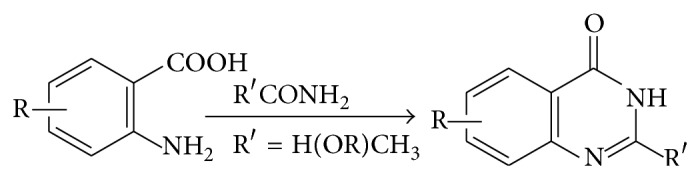


Compound 3 or 4-substituted anthranilic acid when reacted with formamide at 125–130°C gave 3,4-dihydro-4-oxoquinazoline (see Scheme 8).

### 4.2. Grimmel, Guinther, and Morgan's Synthesis

**Scheme 9:**
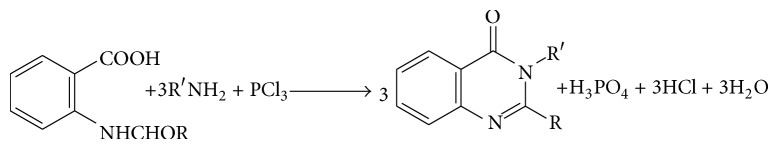


The o-amino benzoic acids, when heated with an amine together with phosphorous trichloride in toluene for two hours, gave 2,3-disubstituted 3,4-dihydro-4-oxoquinazolines (see Scheme 9).

### 4.3. From Isatoic Anhydride

**Scheme 10:**
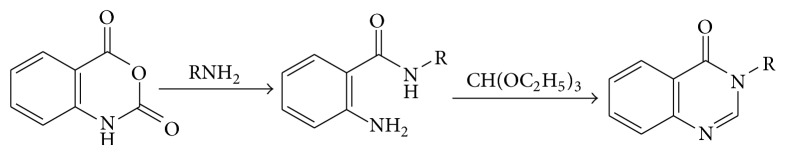


Isatoic anhydride was readily reacted with amines to dihydro-4-oxoquinazolines by refluxing ethyl orthoformate for 1–6 hrs without isolating the intermediate amides (see Scheme 10).

### 4.4. From 3,1,4-Benoxazones (Acylanthranils) and Amines

**Scheme 11:**
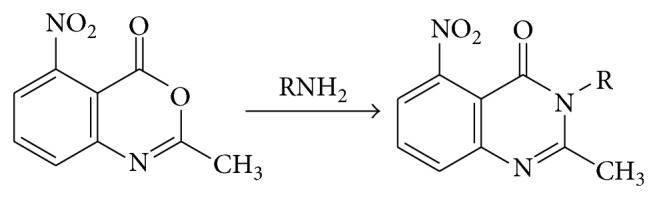


3,1,4-Benoxazones react with amines to give 3,4-dihydro-4-oxoquinazolines. Primary aliphatic amines and anilines react with 2-methyl-5-nitro-4-oxoquinazolines (see Scheme 11).

### 4.5. From Ethyl 2-Acetamido-5-nitrobenzoate

**Scheme 12:**
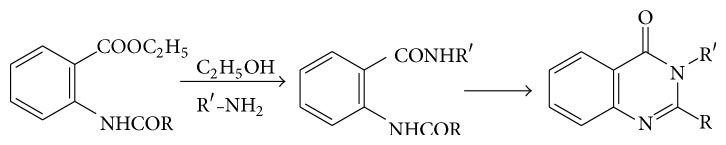


Ethyl 2-acetamido-5-nitrobenzene and alcoholic ammonia when heated gave 3,4-dihydro-methyl-6-nitro-4-oxoquinazoline (see Scheme 12).

### 4.6. Sen and Ray's Synthesis

**Scheme 13:**
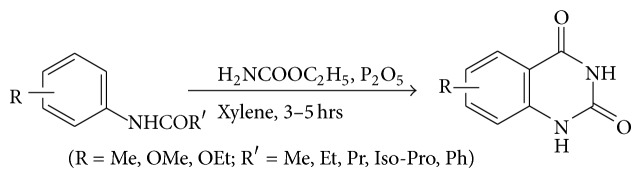


Boiling a solution of normal or isobutyrylanilides with urethane and phosphorous pentoxide in xylene gave 2-propyl and 2-isopropyl-3,4-dihydro-4-oxoquinazolines (see Scheme 13).

## 5. Methods for the Synthesis of Quinazoline and Quinazolinone Derivatives (Benzoylene Urea)

Some methods were reported for the synthesis of quinazolines and quinazolinones are as follows.

### 5.1. From Anthranilic Acid and Urea

**Scheme 14:**
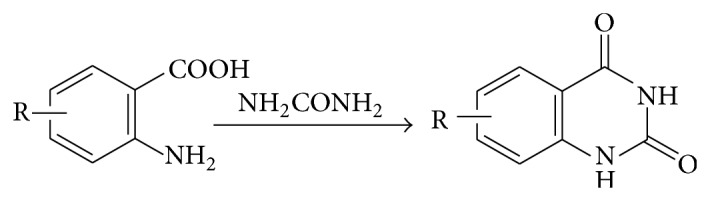


The fusion of anthranilic acid with urea gave 1,2,3,4-tetrahydro-2,4-dioxoquinazoline (see Scheme 14).

### 5.2. From O-Ureidobenzoic Acid

**Scheme 15:**
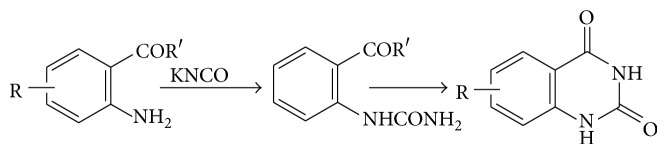


The o-ureidobenzoic acids are prepared from the corresponding anthranilic acid and potassium cyanate. The ureido acids are then easily cyclized to the respective 1,2,3,4-tetrahydro-2,4-dioxoquinazolines by heating with acid or alkali (see Scheme 15).

### 5.3. From O-Ethoxy Carbonylaminobenzoic Esters or Amides

**Scheme 16:**
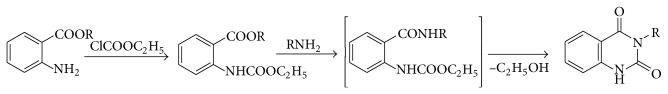


When o-ethoxycarbonylamino benzamide and its 4-methyl derivatives are heated over their melting points, then they lose water and form 1,2,3,4-tetrahydro-2,4-dioxoquinazoline (see Scheme 16).

### 5.4. From Phthalic Acid Derivatives

**Scheme 17:**
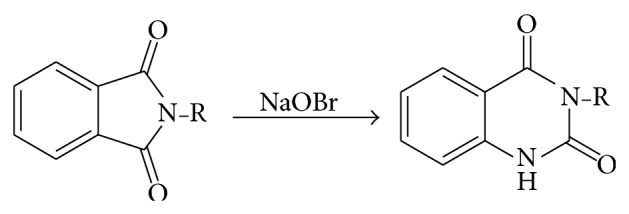


The derivatives of phthalic acid used for the preparation of dioxoquinazoline necessitate rearrangement of the Hoffmann Curties or Lossan type. Reaction of phthalamide or phthalimide, N-methyl, and N-ethyl phthalimide with alkali hypobromite gives the 1,2,3,4-tetrehydro 2,4-dioxoquinazoline (see Scheme 17).

### 5.5. From Isatins

**Scheme 18:**
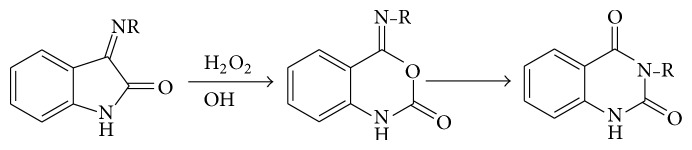


*α*-Isatin oxime rearranges to 1,2,3,4-tetrahydro-2,4-dioxoquinazoline on heating with dilute sodium hydroxide; *β*-imino derivatives of isatin, on the other hand, require oxidation with hydrogen peroxide in alkaline solution in order to form the dioxoquinazoline (see Scheme 18).

### 5.6. From 2-Aminobenzylamine

**Scheme 19:**
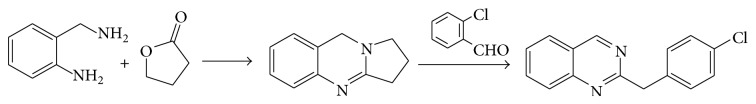


The 2-aminobenzylamine reacts with butyrolactone which involves forming intermediate compound and further condensed with benzaldehyde to give 3-(2-chlorobenzylidene)-1,2,3,9-tetrahydropyrrolo-2-quinazoline (see Scheme 19).

### 5.7. From 2-Azido-4-chlorobenzoic Acid

**Scheme 20:**
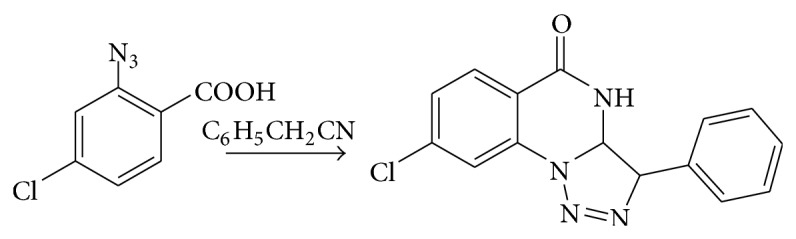


The 2-azido-4-chlorobenzoic acid reacts with benzyl nitrile and formed 7-chloro-3-phenyl-[1,2, 3]triazolo[1,5-a]quinazoline-5-one [9] (see Scheme 20).

Condensation of o-iodobenzaldehydes with amidine hydrochlorides under ligand-free copper catalyzed Ullmann N-arylation conditions afforded the corresponding quinazolines [10]. Treatment of benzoxazine with hydrazine hydrate in ethanol prepared 3-amino-2-phenyl quinazolin-4-(3H)-one, which upon condensation with aldehydes gives the corresponding 3-arylidenoamino derivatives. Cyclization of these derivatives using mercaptosuccinic acid afforded 1,3-thiazolidin-4-one ethanolic acid, which after esterfication with N-hydroxyphthalimide or N-hydroxysuccinamide via acid chlorides produced the respective ethanolic esters [11]. A series of quinoxalin-2(1H)-one-3-hydrazone derivatives were synthesized via condensation of 3-hydrazinoquinoxalin-2(1H)-one with the corresponding ketones under microwave irradiation and gave hydrazones in high yield in less reaction time compared to conventional method [12]. The (hydroxyimino)(2-phenyl(1,2,3,4-tetrahydroquinazolin-2-yl)) methane and (hydroxyimino) (2-(2-thienyl)(1,2,3,4-tetrahydro quinazolin-2-yl)) methane were synthesized by the condensation of 2-(hydroxyimino)-1-phenylethan-1-one and 2-(hydroxyimino)-1-(2-thienyl) ethan-1-one with 2-aminobenzylamine (2-ABA). Complexes of these ligands with Co^3+^ were prepared with a metal: ligand ratio of 1 : 2 [13]. The [4+2]cycloaddition between 2,4-diphenylpyrimidine ortho-quinodimethane and dimethyl acetylenedicarboxylate leads to 2,4-diphenylquinazoline-6,7-dicarboxylate. 2,4-Diphenylfuro [3,4-g]quinazoline-6,8-dione is also obtained by basic hydrolysis of compound, followed by the closure of the resulting diacid in acetic anhydride [14]. A series of triazoloquinazolinones and benzimidazoquinazolinones has been achieved under microwave irradiation by the reaction of aromatic aldehydes with 5-amino-1(H)-1,2,4-triazole (or 2-aminobenzimidazole) and dimedone in DMF [15].

## 6. Biological Importance of Quinazoline Derivatives

The quinazoline and quinazolinone skeleton is frequently encountered in medicinal chemistry. The various substituted quinazolines and quinazolinones are having significant antihypertensive, antineoplastic, antidepressant, and antipsychotic activities whereas some derivatives of quinazoline and quinazolinones are found to be effective agents such as analgesic, antipsychotic, antiarrhythmic, sedative hypnotics, antibacterial, anti-inflammatory, antifungal, antimalarial, anticonvulsant, anticoccidial, anti-Parkinsonism, cancer and other activities [6–8].

### 6.1. Quinazolinones as Anticancer Activity

**Scheme 21:**
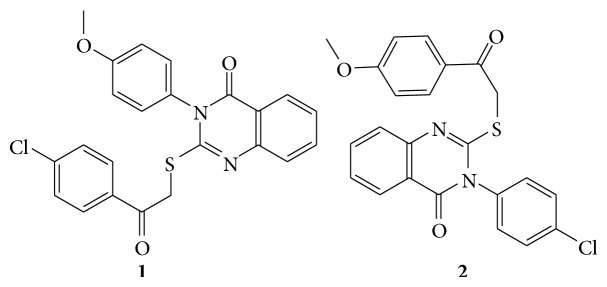


Some new 3-substituted quinazolin-4(3H)-ones and 3,4-dihydro-quinazolin-2(1H)-one derivatives are reported that compounds 2-[2-(4-chlorophenyl)-2-oxo-ethylthio]-3-(4-methoxyphenyl)quinazolin-4(3H) one (**1**) and 3-(4-chlorophenyl)-2-[2-(4-methoxyphenyl)-2-oxo-ethylthio]quinazolin-4(3H)-one (**2**) as broad-spectrum antitumors show effectiveness toward numerous cell lines that belong to different tumor subpanels [16] (see Scheme 21).

**Scheme 22:**
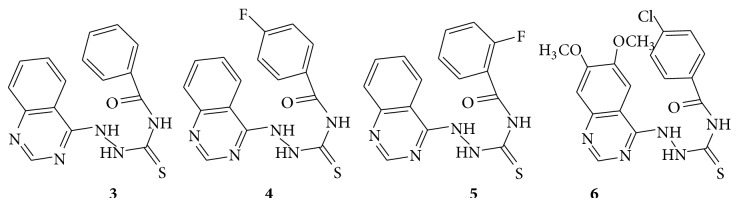


A series of novel quinazoline derivatives (**3**–**6**) containing thiosemicarbazide moiety and evaluate their biological activity as antitumor agents [17]. The therapeutically important candidates are shown in (see Scheme 22).

**Scheme 23:**
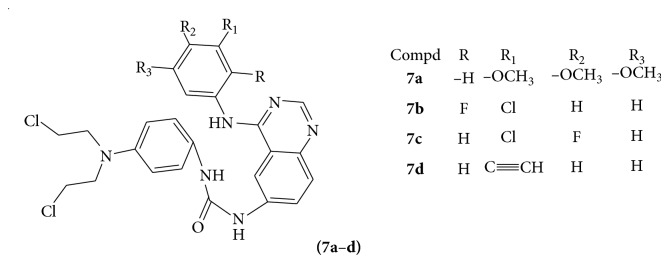


A series of phenyl N-mustard-quinazoline derivatives (**7a**–**d**) were evaluated for their antitumor activity [18] (see Scheme 23).

**Scheme 24:**
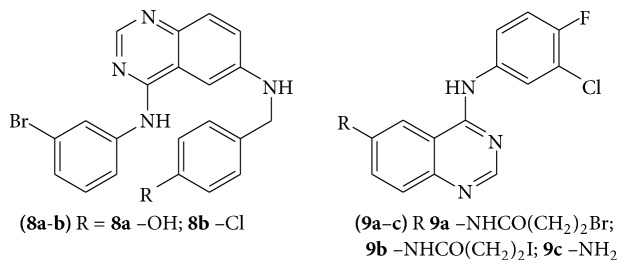


A series of few 4,6 di-substituted-(diaphenylamino)quinazolines derivatives (**8a**-**b**) were evaluated for antitumor activity was considered as potent EGFR inhibitors [19]. A series of quinazoline derivatives (**9a**–**c**) were evaluated for their function as EGFR inhibitors by applying radioiodination. All these compounds were further evaluated for potential SPECT activity for molecular imaging of breast cancer [20] (see Scheme 24).

**Scheme 25:**
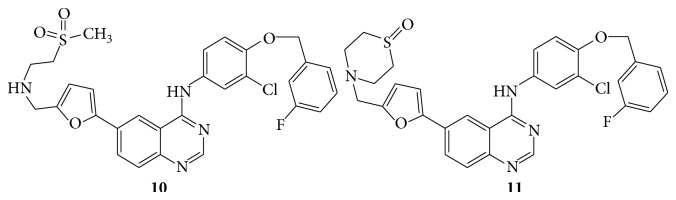


**Scheme 26:**
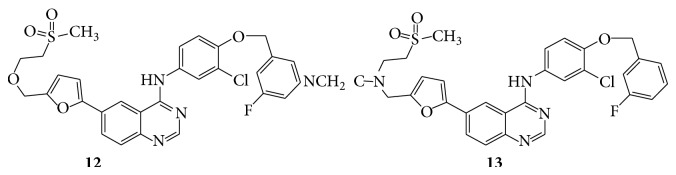


A series of novel 6-furanylquinazoline derivatives (**10**–**13**) were subsequently evaluated for their biological activity as a potent ErbB-1/ErB-2 tyrosine kinase inhibitor [21] (see Schemes 25 and 26).

**Scheme 27:**
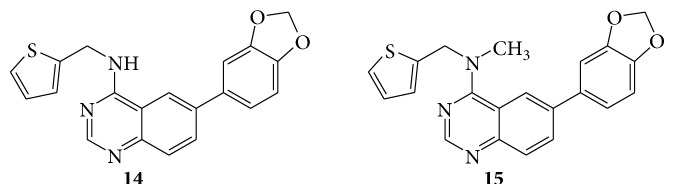


**Scheme 28:**
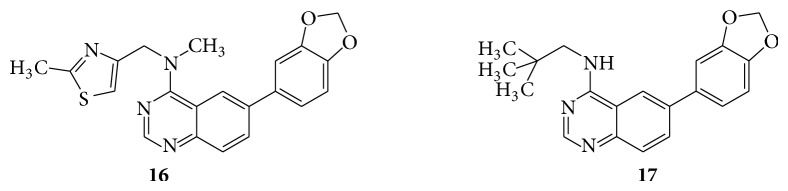


A series of quinazoline derivatives (**14**–**17**) were evaluated for their activity as potent inhibitors of specific isoforms of Cdc2-like kinases (Clk) and dual specificity tyrosine-phosphorylation regulated kinases (Dyrk) [22] (see Schemes 27 and 28).

**Scheme 29:**
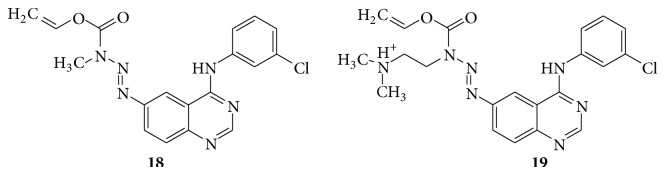


New combi-triazenes (**18**,** 19**) for targeting solid tumors express the epidermal growth factor receptor (EGFR) or its closest homologue HER2 (see Scheme 29).

**Scheme 30:**
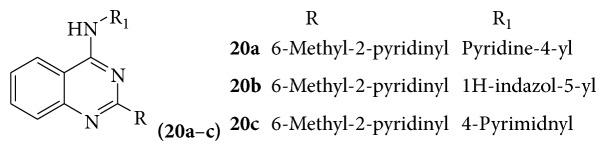


A series of novel quinazoline derivatives (**20a**–**c**) showed potent ALK5 inhibitory activity [23] (see Scheme 30).

**Scheme 31:**
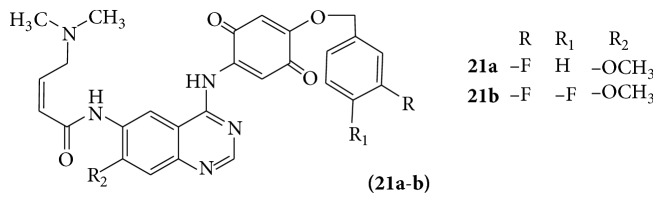


Quinazoline-based (**21a-b**) anticancer molecule is dual irreversible kinase inhibitors [24] (see Scheme 31).

**Scheme 32:**
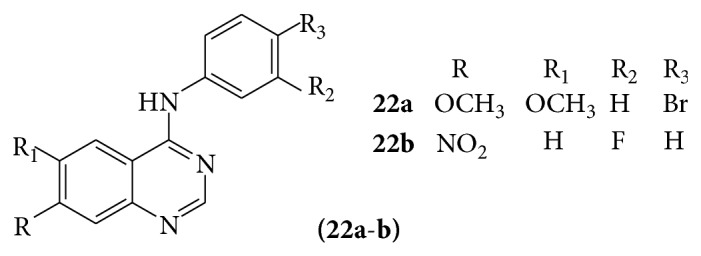


A series of quinazoline derivatives (**22a-b**) were evaluated for their biological activity against tyrosine kinase (EGFR) [25] (see Scheme 32).

**Scheme 33:**
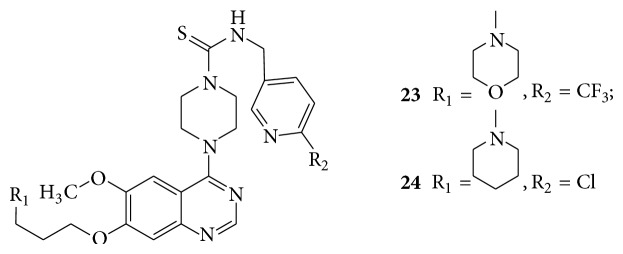


**Scheme 34:**
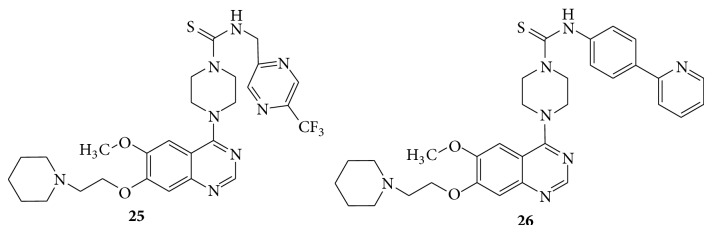


A series of 4-piperazin-1-yl-quinazoline template based aryl and benzyl thiourea derivatives (**23**–**26**) showed potent, selective, and orally bioavailable antagonist of platelet-derived growth factor (PDGF) receptor [26] (see Schemes 33 and 34).

**Scheme 35:**
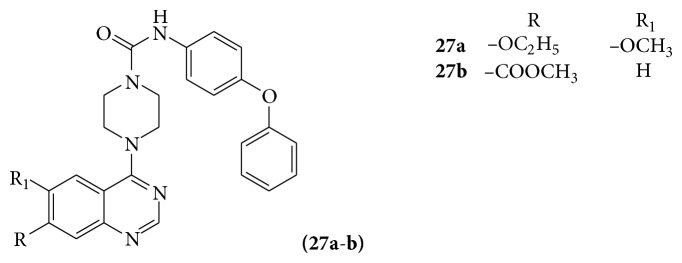


A series of 4-[4-(N-substituted(thio)carbamoyl)-1-piperazinyl]-6,7-dimethoxyquinazoline derivatives (**27a-b**) were evaluated for their potential antagonizing activity against Platelet-Derived Growth Factor Receptor (PDGF) [27] (see Scheme 35).

**Scheme 36:**
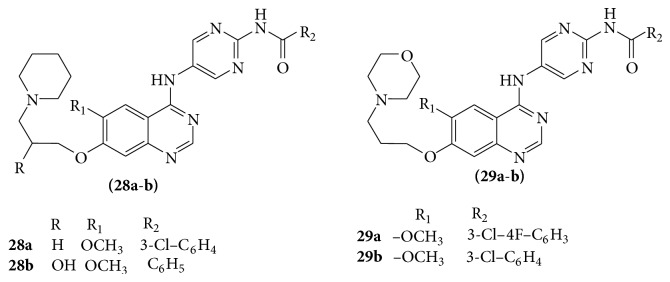


A series of quinazoline derivatives (**28a-b** and** 29a-b**) showed potent inhibitory activity against Aurora kinase [28] (see Scheme 36).

**Scheme 37:**
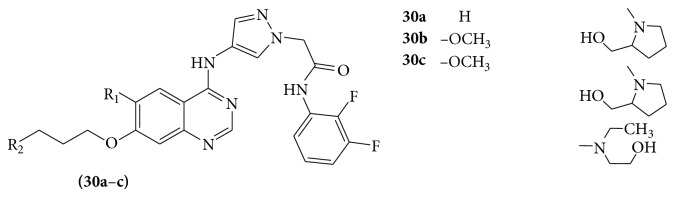


A series of 1-acetanilide-4-aminopyrazole substituted quinazoline derivatives (**30a**–**c**) were subsequently evaluated for their inhibitory activity against Aurora B kinase as potent antitumour agents [29] (see Scheme 37).

**Scheme 38:**
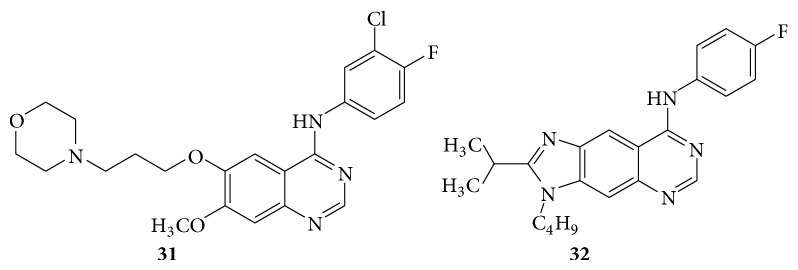


Some quinazolines were evaluated as antitumor agents, the biological activity of some 2,3-di-substituted 8-arylamino-3H-imidazo[4,5-g]quinazoline derivative (**31** and** 32**) as a potent antitumor agent. Compound** 32** possessed the highest activity on the A549 cell line [30] (see Scheme 38).

**Scheme 39:**
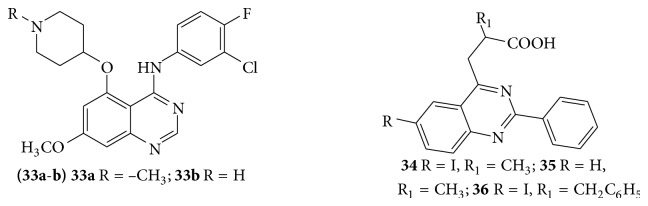


A series of novel C-5 substituted anilinoquinazoline derivatives and evaluated their activity as an inhibitor of epidermal growth factor receptor tyrosine [31]. Few novel 4,6-disubstituted quinazoline derivatives (**33**–**36**) showed good anti-inflammatory and anticancer activity (cytotoxic) against U937 leukemia cell lines [32] (see Scheme 39).

**Scheme 40:**
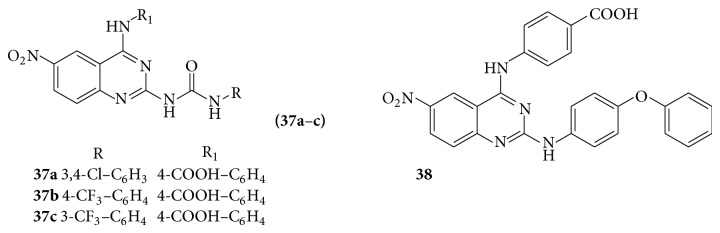


A series of quinazoline derivatives (**37** and** 38**) have strong inhibition on human Pin1 [33] (see Scheme 40).

**Scheme 41:**
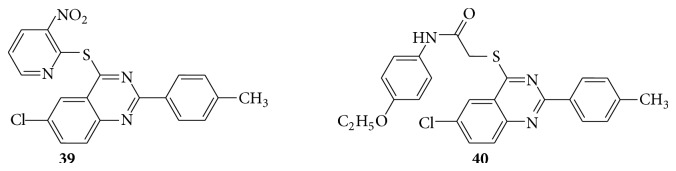


**Scheme 42:**
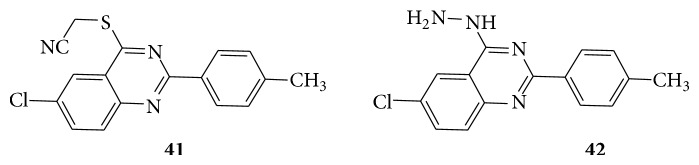


A series of quinazoline derivatives (**39**–**42**) were evaluated for their biological activity as potential antitumor agents [34] (see Schemes 41 and 42).

HEPG2 human liver cell line was proved to be sensitive toward compounds** 39**,** 40**, and** 41** with IC_50_ concentration range of 4.17–5.99 *μ*g/mL. Regarding HELA cervix cell line, higher sensitivity was observed with compounds** 39**,** 41**, and** 42** with IC_50_ concentration range of 3.56–5.39 *μ*g/mL. With regard to broad-spectrum antitumor activity, compounds** 42**,** 41,** and** 39** showed IC_50_ of 3.35–5.59 *μ*g/mL against the three cell lines.

**Scheme 43:**
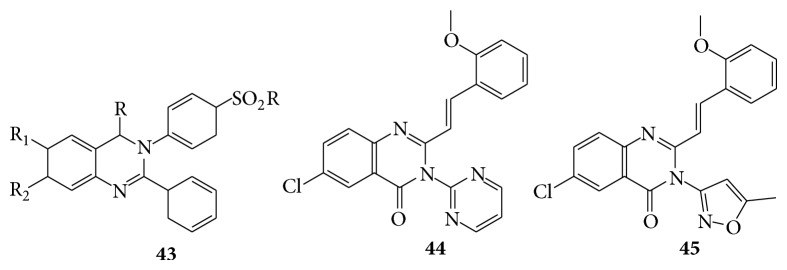


Microwave-assisted synthesis and the SAR studies of modified 9-oxo-thia-zolo[5,4-f] quinazoline-2-carbonitriles allowed identification of new amidine and imidate derivatives as potent and dual CDK1/GSK-3 inhibitors. Combination of lead optimization and molecular modeling studies allowed a dual CDK1/GSK-3 inhibitor with submicromolar values [35]. Novel 2,3-disubstituted quinazoline-4(3H)-ones (**43**) were screened for cytotoxicity and for antiviral activity against influenza A [36]. The 6-Arylbenzimidazo [1,2-c]quinazoline derivatives were act as a tumor necrosis factor alpha (TNF-*α*) production inhibitors. These compounds were tested for their* in vitro* ability to inhibit the lipolysaccharide (LPS) induced TNF-*α* secretion in the human promyelocytic cell line HL-60. The compound 6-Phenyl-benzimidazo [1,2-c]quinazoline, coded as G1, resulted as the most potent inhibitor and with no significant cytotoxic activity. Thus, 6-arylbenzimidazo [1,2-c]quinazoline derivatives may have a potential as anti-inflammatory agents [37]. Docking studies of few synthesized 6,7-dialkoxy-4-anilinoquinazoline derivatives which showed EGFR-TK inhibitory activity were conducted [38]. The 3-(3-methylisoxazol-5-yl) and 3-(pyrimidin-2-yl)-2 styrylquinazolin-4(3H)-ones (**44**,** 45**) were prepared by refluxing in acetic acid the corresponding 2-methylquinazolinones with the benzoic aldehyde and tested for their* in vitro* antileukemic activity against L-1210 (murine leukemia), K-562 (human chronic myelogenous leukemia), and HL-60 (human leukemia) cell lines showing in some cases good activity [39] (see Scheme 43).

### 6.2. Quinazolinones as Antibacterial Activity

**Scheme 44:**
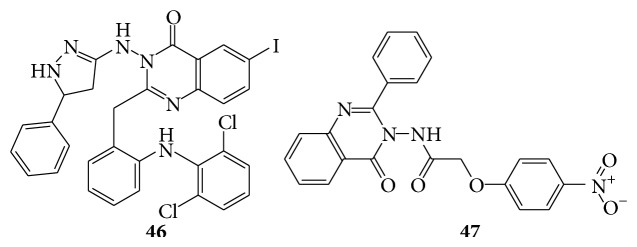


**Scheme 45:**
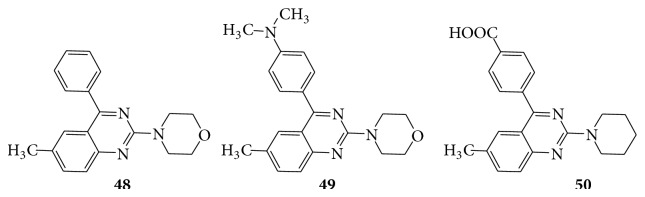


A series of new 2-[2-(2,6-dichlorophenyl)amino]phenyl methyl-3-[(5-substitutedphenyl)-1,5-dihydro-1H-pyrazol-3-yl-amino]-6-iodoquinazolin-4(3H) ones compounds (**46**) were tested for their antibacterial activity* in vitro* by measuring zone of inhibition in mm against different strains like two gram positive bacteria, namely,* Staphylococcus aureus* and* Bacillus subtilis,* and two gram negative bacteria, namely,* Escherichia coli* and* Certium* at two different concentrations 100 *μ*g/mL and 50 *μ*g/mL [40]. Quinazolinone derivatives (DK-1, DK-2, DK-3, DK-4, DK-5, DK-6, and DK-7) by treating 2-chloro-N-(4-oxo-2-phenylquinazolin-3(4H)-yl)acetamide with the different substituted phenols. The synthesized compounds were evaluated for antibacterial activity by cup plate method by measuring inhibition zone. The compound DK-2 (**47**) showed more potent antibacterial activity than the standard drug ampicillin [41]. A series of quinazolines derivatives were evaluated for their biological activity on various bacterial cultures [42] (see Schemes 44 and 45).

**Scheme 46:**
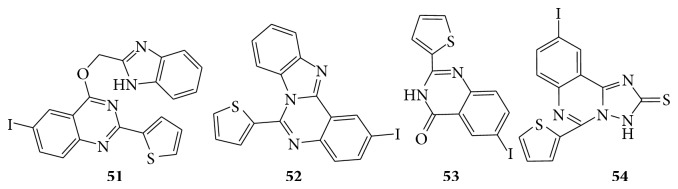


Compounds** 49** and** 50** showed comparative activity against* K. pneumoniae* as compared to ciprofloxacin. Compound** 48** exhibited greater activity against* S. sonnei, E. faecalis,* and* P. aeruginosa* as compared to ciprofloxacin. A series of some novel substituted iodoquinazoline derivatives are evaluated for their antimicrobial activity [43]. Compounds** 52** and** 53** showed remarkable activity towards the gram negative bacteria* E. coli,* whereas compounds** 51**,** 52**, and** 54** showed potent activity against* S. aureus, B. subtilis, S. Cerevisiae,* and* C. albicans* (see Scheme 46).

**Scheme 47:**
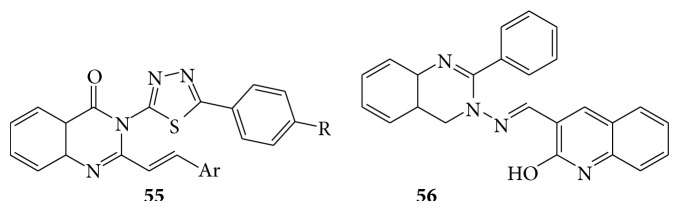


**Scheme 48:**
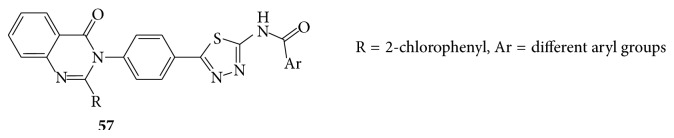


The 3-[5-(4-substituted phenyl)-1,3,4-thiadiazole-2-yl]-2-styryl quinazoline-4(3H)-ones (**55**) reported their antibacterial and antifungal activity [44]. The 6,7,8,9-tetrahydro-5(H)-5-nitrophenylthiazolo[2,3-b]-quinazolin-3(2H)-one derivatives showed antimicrobial activity [45]. The 3-[(2-hydroxy-quinolin-3-ylmethylene)-amino]-2-phenyl-3H-quinazolin-4-one (**56**) and its metal (II) complexes were reported for their antimicrobial activity [46]. Some quinazoline derivatives (**57**) act as potential antimicrobial agents [47] (see Schemes 47 and 48).

Condensing 2-methyl/phenyl/chloro methyl disubstituted benzooxazine-4-one and 1-(2-amino ethyl)-4-substituted benzylidene-2-phenyl-1H-Imidazoles-5(4H)-one gave imidazolo-quinazoline-4-one derivatives. These compounds have shown promising antibacterial and antifungal activity [48]. The antibacterial activities of substituted quinazolines against bacterial strains* E. coli, P. aeruginosa, B. subtilis,* and* S. aureus* were investigated. The sensitivity of the gram positive bacteria to the tested quinazolines was higher than that of gram negative bacteria. The most effective of quinazoline structure series were condensed [1,2,4]triazoloquinazolines and 10H-[1,2,4]triazino[5,4-b]quinazolin-10-ones [49]. The 6-bromo-2-alkyl/aryl-3{[phenyl(phenyldiazenyl)methylene]amino}quinazolin-4(3H)-ones were exhibited antimicrobial activities [50].

### 6.3. Quinazolinones as Antifungal Activity

Octahydroquinazoline (**58**) was obtained by a modification of the Biginelli reaction with phenacyl bromide and bromo malononitrile to furnish thiazolo [2,3-b] quinazoline and they found the interaction of compound with formamide, formic acid, and phenyl isothiocyanate yielded the corresponding pyrimidino thiazolo [2,3-b] quinazolines and exhibited antifungal activity against* Candida albicans* [51].

**Scheme 49:**
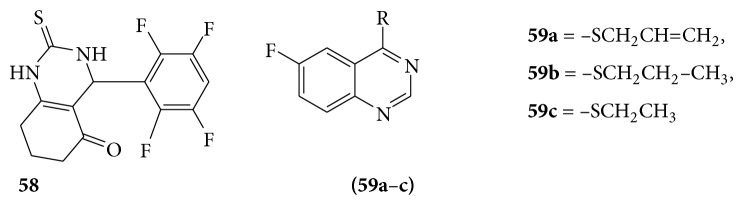


A series of few novel S-substituted-6-fluoro-4-alkyl (aryl) thioquinazoline derivatives (**59a**–**c**) were evaluated for their pharmacological activity as antifungal [52] (see Scheme 49).

All of these compounds exhibited good antifungal activity, especially compound** 59c**, having a wide spectrum of bioactivity; it shows potent inhibitory activity on the growth of most of the fungi with EC_50_ values ranging from 8.3 to 64.2 *μ*g/mL.

### 6.4. Antitubercular Activity

Some quinazolinones were reported as potent chemotherapeutic agents in the treatment of tuberculosis (TB). For example 3-aryl-6,8-dichloro-2H-1,3-benzoxazine-2,4(3H)-diones (**60**) and 3-arylquinazoline-2,4(1H,3H)-diones (**61**) are as anti-TB agents and quinazolinone derivatives as anti-TB agents [4].

**Scheme 50:**
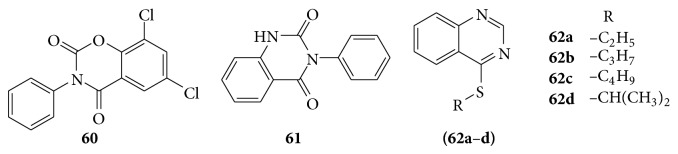


A series of 2-alkylthio-6-iodo-3-substituted-quinazolin-4-one derivatives were screened for their* in vitro* anti-TB activity against* Mycobacterium tuberculosis* strain HRv [53]. A series of quinazoline derivatives (**62a**–**d**) were evaluated for their pharmacological activity as anti-TB [54] (see Scheme 50).

1,4-Disubstituted 3-[3′-(2′-phenyl-4′-oxo-quinazolinyl)]-2-azetidinones showed antifertility activity [55]. 6-substituted-2-phenyl-3-(5-substituted mercapto-1,3,4-thiadiazole-2-yl)quinazoline-4-(3H)-ones showed anti-TB activity [56]. Most of the synthesized compounds exhibited anti-TB activity against the strains of* Mycobacterium tuberculosis, M. avium, M. fortuitum, M. kansasii, *and* M. intracellulare*. The modification process with various hydrophobic chains clearly suggests the existence of hydrophobic pocket in the active site of the target of various strains of* Mycobacterium* spp., which eventually raise the therapeutic efficacy.

### 6.5. Quinazoline as Antiviral Agents

**Scheme 51:**
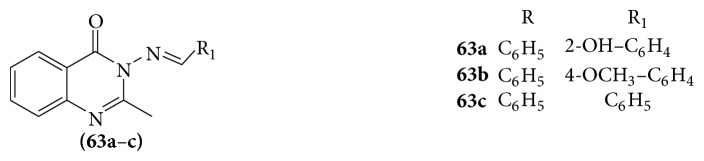


A series of Schiff bases of some 2-phenyl quinazoline-4(3)H-one derivatives are evaluated for their activity as antiviral agents [57] (see Scheme 51).

**Scheme 52:**
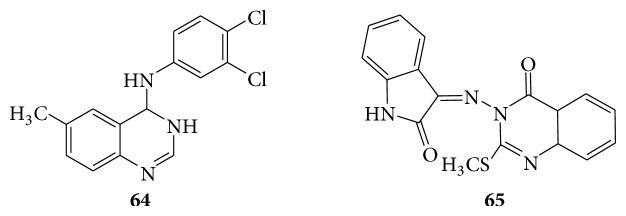


Compound** 63a** exhibited antiviral activity against herpes simplex virus-1 (KOS), herpes simplex virus-2(G), herpes simplex virus-1 (TK- KOS ACV), and vaccinia virus in HEL cell culture at selectivity index of 100, 100, 100, and 125, respectively, whereas cytotoxicity was observed at 100 *μ*g/mL. Compounds** 63b** and** 63c** demonstrated good activity against herpes simplex virus-1 (KOS), herpes simplex virus-2 (G), and vaccinia virus. The protein kinase inhibitory activity and anticytomegaloviral activity showed few quinazoline (**64**) compounds [58]. Quinazolinones act as anti-HIV activity whereas compounds 3-amino-2-methyl mercaptoquinazolin-4(3H)-one (**65**) were synthesized by condensing the acidic imino group of isatin with formaldehyde and secondary amines and evaluated for anti-HIV activity against HIV-1 III B in MT-4 cells [59] (see Scheme 52).

### 6.6. Quinazolinones as Antimutagenic Activity

The (S)-4-aminoquinazoline alcohols (**66**) were prepared from enantiomerically pure from (S)-quinazolinone alcohols. Mutagenic and antimutagenic properties of the (S)-4-aminoquinazoline alcohols were investigated by using* Salmonella typhimurium* and* E. coli* WP2uvrA tester strains at 0.01, 0.1, and 1 lg/plate concentrations. (S)-4-aminoquinazoline alcohols were found to be genotoxically safe at the tested concentrations. Among the tested (S)-4-aminoquinazoline alcohols, the best antimutagenic activity was obtained with a methyl derivative at 0.1 *μ*g/plate dose [60].

### 6.7. Quinazolinones as Anticoccidial Activity

**Scheme 53:**
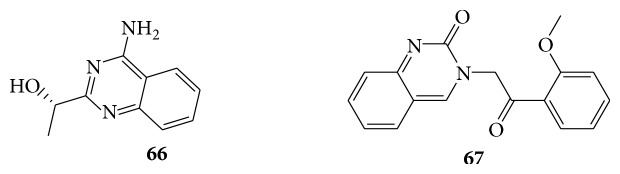


**Scheme 54:**
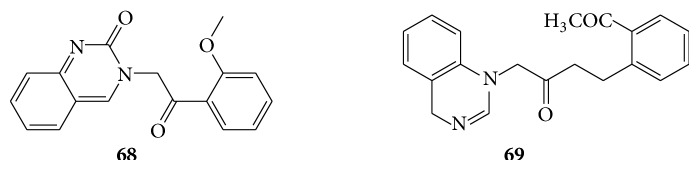


A series of 3-(2-(2-methoxyphenyl)-2-oxoethyl) quinazolinone derivatives (**67**) are anticoccidial agents by modifying the quinazoline ring of febrifugine against* Eimeria tenella* in the chicken at a dose of 9 mg/kg. 3-(2-(2-Methoxyphenyl)2-oxoethyl) quinazolinone derivatives (**68**) possess high anticoccidial activity and may serve as a lead compound for the development of anticoccidial drugs in the future [61]. A series of 4-(2-methoxyphenyl)-2-oxobutylquinazoline (**69**) derivatives are reported for their anticoccidial activity [62] (see Schemes 53 and 54).

### 6.8. Anti-Inflammatory and Analgesics Agents

**Scheme 55:**
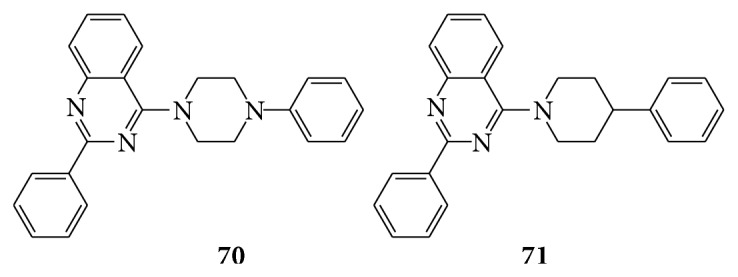


A series of quinazoline derivatives (**70**,** 71**) showed potent analgesic and anti-inflammatory activity. All these compounds demonstrated potent activity as anti-inflammatory analgesic more than the reference compound indomethacin [63] (see Scheme 55).

**Scheme 56:**
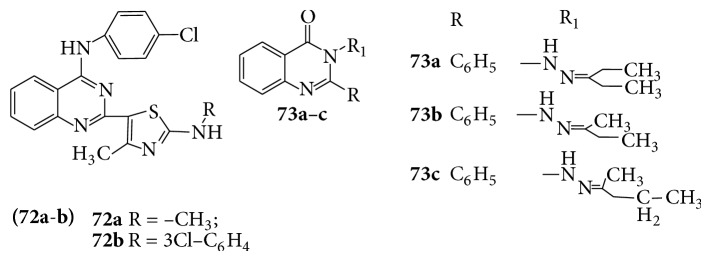


A series of novel 2-(2,4-disubstituted-thiazole-5-yl)-3-aryl-3H-quinazoline-4-one (**72**) derivatives which became good inhibitors of NF*κ*B and AP-1 mediated transcription activation [64]. A series of 3-phenyl-2-substituted-3H-quinazolin-4-one (**73a**–**c**) derivatives were evaluated for their pharmacological activity as analgesic and anti-inflammatory agents [65] (see Scheme 56).

**Scheme 57:**
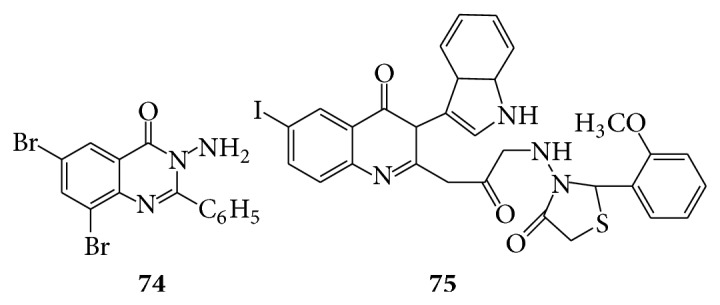


A series of some novel 2,3-disubstituted quinazolinone derivatives by condensing 2-methyl/2-phenyl/6-bromo-2-methyl/6-bromo-2-phenyl/6,8-dibromo-2-methyl/6,8-dibromo-2-phenyl benzoxazines with compounds containing amino group were evaluated for their analgesic activity and they reported that compound (**74**) shows promising analgesic activity compared to standard drug Diclofenac sodium [66]. Various 2-(substituted phenyl methylene imino) amino acetyl methylene-3-(2′-substituted indol-3′-yl)-halo substituted-4(3H) quinazolinone and 2-(substituted phenyl amino methylene acetyl-4′-oxo-1′-thiazolidinyl-3-(2′′-substituted indol-3′′-yl)4(3H)-quinazolinones reported that compound (**75**) exhibited good anti-inflammatory activity [67] (see Scheme 57).

**Scheme 58:**
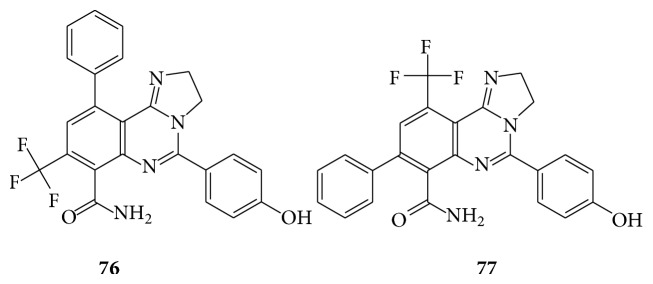


A series of novel 8/10-trifluoromethyl-substituted-imidazo [1,2-c] quinazolines are evaluated* in vivo* for their anti-inflammatory activity and* in silico* to recognize the hypothetical binding motif with the cyclooxygenase isoenzymes (COX-1 and COX-2) employing GOLD (CCDC, 4.0.1 version) software and found that compounds (**76** and** 77**) show good anti-inflammatory activity against standard: indomethacin [68] (see Scheme 58).

A series of 5-(4-chlorophenyl)-9-iodo-3-substituted-l,2,4-triazolo[4,3-c]quinazoline and 2-(4-chlorophenyl)-6-iodo-4-substituted-quinazoline were evaluated as anti-inflammatory agents. The result revealed that some compounds have activity comparable to indomethacin [69]. A number of substituted oxoquinazolines were reported for their analgesic and antibacterial activity [70]. A series of novel 3-(6-substituted-1,3-benzothiazole-2-yl)-2-[{(4-substituted phenyl)amino} methyl]quinazolines-4(3H)-ones and quinazolines-4-one derivative were investigated for their anti-inflammatory and antibacterial activity [71]. A series of some 2-[(E)-2-furan-2-yl-vinyl]-quina-zolin-4(3H)-ones incorporated into pyrazoline, isoxazoline, pyrimidine, or pyrimidine-thione ring systems at position 3 of the quinazoline ring. These compounds showed antimicrobial activity and anti-inflammatory effect of [72].

### 6.9. Quinazoline as Antidepressant and Anticonvulsant

**Scheme 59:**
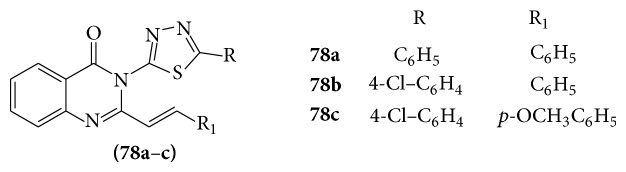


A series of novel 3-[5-substituted 1,3,4-thiadiazole-2-yl]-2-styryl quinazoline-4(3H)-ones (**78a**–**c**) derivatives were evaluated for their activity as antidepressant agents [73] (see Scheme 59).

All the compounds showed anticonvulsant activity in MES screen; however, compound** 78a** showed potency similar to standard drug (phenytoin, carbamazepine) without any neurotoxicity.

**Scheme 60:**
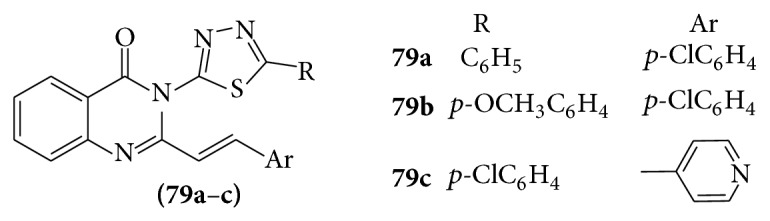


A series of some novel 3-[5-substituted 1,3,4-thiadiazole-2-yl]-2-styryl quinazoline-4(3H)-one.

(**79a**–**c**) derivatives were evaluated for their activity as CNS depressant and anticonvulsant agents [74] (see Scheme 60).

Compounds with the above substituents showed potent CNS depressant activity. Compound** 79a** showed anticonvulsant activity at 0.5 and 4 h in different test models, whereas** 79c** showed anticonvulsant activity at 4 h in MES screen and at 0.5 and 4 h in subcutaneous PTZ screen.

**Scheme 61:**
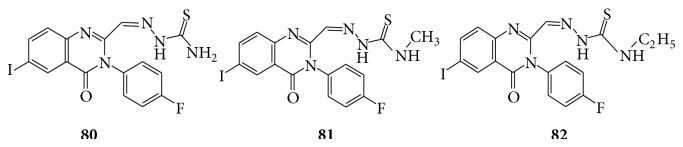


The 3-aryl-4(3H)-quinazolinone-2 carboxaldehydes, their corresponding Schiff's base, and thiosemicarbazone derivatives reported (**80**–**82**) as anticonvulsants [75] (see Scheme 61).

**Scheme 62:**
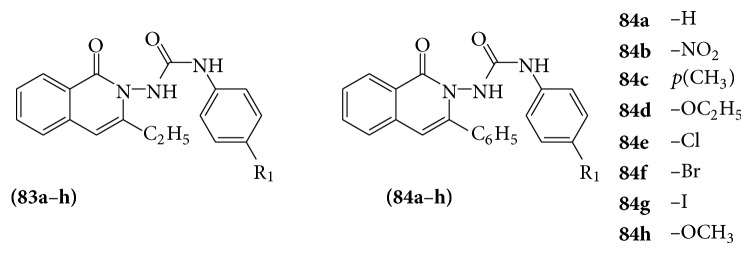


Several 1-(4-substitutedphenyl)-3-(4-oxo-2-phenyl/ethyl-4H-quinazolin-3-yl)-urea derivatives (**83a**–**h** and** 84a**–**h**) were screened for their anticonvulsant activity by MES and scPTZ-induced seizure models in mice and found that these compounds were active in the MES screen whereas some compounds were found to be active in the scPTZ screen [76] (see Scheme 62).

**Scheme 63:**
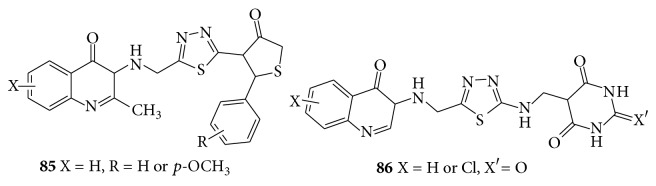


Two regioisomer series, 2-(3-ethyl-4(3H)-quinazolinone-2-ylmercaptoacetylhydrazono)-3-alkyl/3-aryl-5-methyl-4-thiazolidinones (**85**) and 2-arylimino-3-(3-ethyl-4(3H)-quinazolinone-2-yl mercaptoacetylamino)-5-methyl-4-thiazolidinones were reported for their anticonvulsant activity [77]. Substituted quinazolinonyl-2-Oxo/thiobarbituric acid derivatives showed anticonvulsant activity against MES and scPTZ models [78]. A series of halogenated derivatives, 3-methyl, 3-ethyl and 3-phenyl-6-mono, and 6,8-disubstituted-3H-quinazolin-4-one derivatives exhibited anticonvulsant activity and phenobarbitone sodium was used as a reference drug [79]. Some thiadiazolyl and thiazolidinonyl quinazoline-4(3H)-ones (**86**) screened them for anticonvulsant activity against MES induced convulsions in animal models [80] (see Scheme 63).

**Scheme 64:**
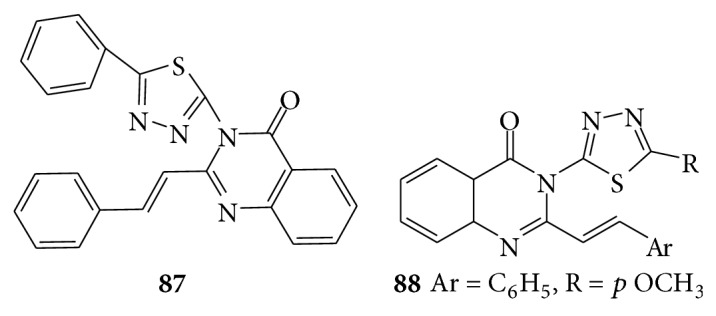


A series of novel 3-[5-substituted phenyl-1,3,4 thiadiazole-2-yl]-2-styryl quinazoline-4(3H)-one is screened for antidepressant activities with the help of the forced swim pool method and found that compound (**87**) was most active against antidepressant activity [73]. The 3-[5-substituted-1,3,4-thiadiazole-2-yl]-2-styryl quinazoline-4(3H)-ones (**88**) and their antidepressant activity were screened with the help of forced swim pool method [73] (see Scheme 64).

### 6.10. Quinazolinones as Antimalarial Agents

**Scheme 65:**
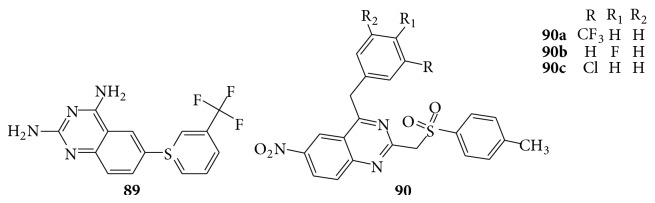


The 2,4-diamino-6-[(aryl)thio]quinazoline compounds were known to their antimalarial properties wherein the 4-amino group was replaced by hydrazine and hydroxyamino moieties and they found that such changes reduce markedly the antimalarial properties of this series. The compound (**89**) was tested against a normal drug-sensitive strain of* Plasmodium berghei* in mice by the parenteral route [81]. A series of quinazoline derivatives (**90**) were evaluated for their antiplasmodial activity [82] (see Scheme 65).

**Scheme 66:**
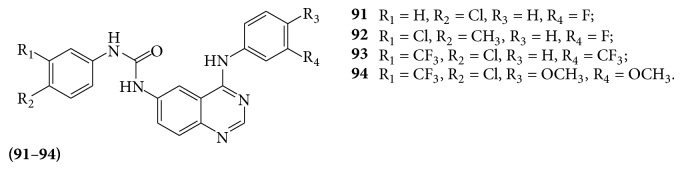


Compounds** 90a** and** 90c** show a high potential activity (resp., W2 IC_50_ values = 0.95 and 1.3 *μ*M) in comparison with chloroquine and doxycycline. A series of new 6-ureido-4-anilinoquinazolines (**91**–**94**) were evaluated for their potent activity as antimalarial agents [83] (see Scheme 66).

**Scheme 67:**
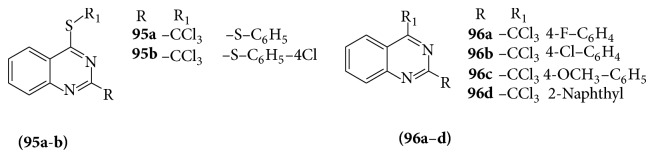


A series of 4-thiophenoxy-2-trichloromethyquinazolines derivatives (**95a-b**) were evaluated for their antiplasmodial activity against the human malarial parasite* Plasmodium falciparum* was determined [84]. Compounds** 95a** and** 95b** showed good activity against K1* Plasmodium falciparum* (IC_50_ = 1.9 *μ*M and 0.9 *μ*M, resp.), whereas IC_50_ value of chloroquine is 0.5 *μ*M. A series of (**96a**–**d**) in 4-aryl-2-trichloromethylquinazolines (**96a**–**d**) were evaluated for their antiplasmodial activity [85] (see Scheme 67).

Compounds with the above substituents exhibited favourable antiplasmodial activity on THP1 and HepG2 human cell lines.

### 6.11. Quinazolinones as Antioxidant Activity

**Scheme 68:**
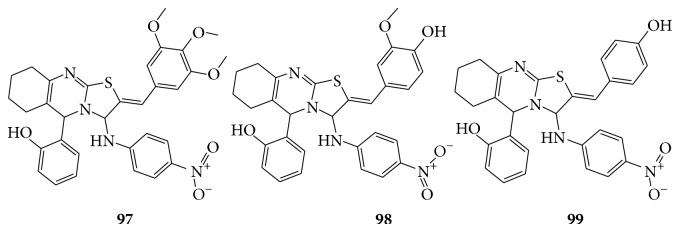


A series of novel thiazolo quinazoline derivatives by condensation of different aromatic aldehydes with 4-nitro aniline are screened for antioxidant activity by DPPH radical assay, nitric oxide scavenging activity, and hydrogen peroxide scavenging activity and reported that synthesized compounds (**97**–**99**) were found to be the most potent antioxidant activity [86] (see Scheme 68).

### 6.12. Antileishmanial Agents

**Scheme 69:**
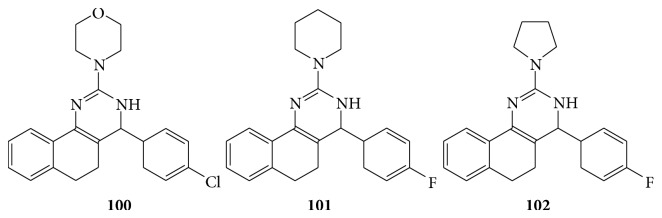


Compounds of both synthetic and natural origin comprising a diverse group of chemical structure have been reported as antileishmial agents. These include mostly nitrogen heterocyclic such as quinolines, purine, pyrimidine, acidine, phenothiazines, bisbenzamides, pyrazolol, pyridine, benzothiazole, and imidazolines [87]. The 4-(substituted-benzylidine)-2-substituted-5,6-dihydrobenzo[h]quinazoline and 4-(substituted benzylidine)-2-substituted-3,4,5,6-tetrahydrobenzo[h]quinazoline from 2-(substituted-benzylidine)tetralone-1 and several substituted guanidine sulfates are evaluated for their* in vitro* antileishmanial activity and they reported that compounds (**100**–**102**) show promising antileishmanial activity against* Leishmania donovani* [88] (see Scheme 69).

### 6.13. Quinazoline as Neuroprotective Agents

**Scheme 70:**
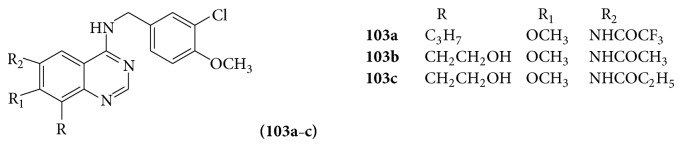


Few quinazoline derivatives (**103a**–**c**) were evaluated for their activity as potent and highly selective PDE5 inhibitors to be employed for male erectile dysfunction [89] (see Scheme 70).

### 6.14. Quinazoline as Antiobesity Agents

**Scheme 71:**
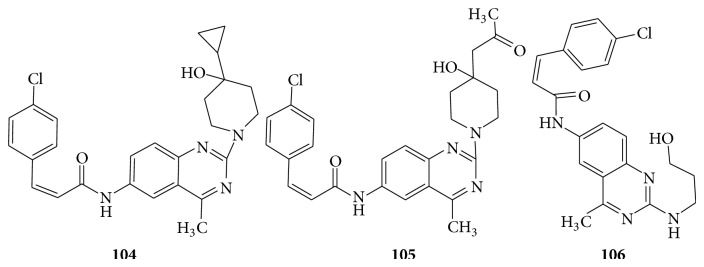


A series of quinazoline derivatives (**104**–**106**) are to be considered as an antagonist for melanin concentrating hormone receptor 1 (MCHR1) [90] (see Scheme 71).

### 6.15. Antihypertensive Agents

A series of 3-benzyl-2-substituted-3H-[1,2,4]triazolo[5,1-b]quinazolin-9-ones have been synthesized by the cyclocondensation of 3-amino-2-benzylamino-3H-quinazolin-4-one. The compounds were evaluated for their* in vivo* antihypertensiveactivity. All the test compounds exhibited significant antihypertensive activity; 3-benzyl-2-methyl-3H-[1,2,4]triazolo[5,1-b] quinazolin-9-one exhibited antihypertensive activity more than the reference drug prazocine [91].

### 6.16. Anti-H_1_-Antihistaminic Agents

A series of 1-substituted-4-benzyl-4H-[1,2,4]triazolo[4,3-a]quinazolin-5-ones were synthesized by the cyclization of 2-hydrazino-3-benzyl-3H-quinazolin-4-one. When tested for their* in vivo* H_1_-antihistaminic activity on guinea pigs, all the test compounds protected the animals from histamine induced bronchospasm significantly [92].

### 6.17. Quinazoline as Antiprotozoan Agents

A series of quinazoline derivatives (**107a**-**b**) were evaluated for their activity as a potent inhibitor of trypanosoma cruzi dihydrofolate reductase [93] (see Scheme 72).

## 7. Discussion

Heterocyclic compound containing quinazoline and quinazolinone nucleus plays most important role in the field of medicinal chemistry. It shows wide range of activities for medication purpose. A large number of quinazoline compounds have been synthesized and evaluated for their different biological activities. Some marketed quinazoline and quinazolinone nucleus containing drugs have different types of pharmacological activities. The quinazoline and quinazolinone based pharmaceuticals are becoming very important class of therapeutic agents and are likely to replace many obtainable organic based pharmaceuticals in the very near future. The quinazoline and quinazolinone based pharmaceuticals will be created on a large scale by different research development processes and will become available commercially for therapeutic uses. The biological profiles of this new generation of quinazoline and quinazolinone represent much progress with regard to the older compounds. This study gets an efficient way of understanding about the target pharmacophore relationship which can further aid the process of drug design developments. This study may also accelerate the designing processes to generate a larger number of therapeutically active molecules. The molecular treatment of potentially lead molecules is still a major line of approaches for the discovery and development of new drug molecules [94–99]. Combination of two or more moieties into one is a general procedure of handling and this can probably result in the raise of biological activities and deduction of untoward side effects.

## 8. Conclusion

The various structural modifications around the fused ring of quinazoline and quinazolinone subsequently evaluate are for their usefulness in treating various disease conditions. Quinazoline and quinazolinone, being the central body of the pharmacophore, hold different types of substituent. Based on their various physicochemical properties, they exerted a diversified range of therapeutic efficacy. Thus we can conclude that this review will definitely provide the researchers with a thorough understanding of the structure activity relationship study, which further helps in designing good large number of quinazoline and quinazolinone compounds with a strong impact in curing many fatal disorders.

## Figures and Tables

**Scheme 72:**